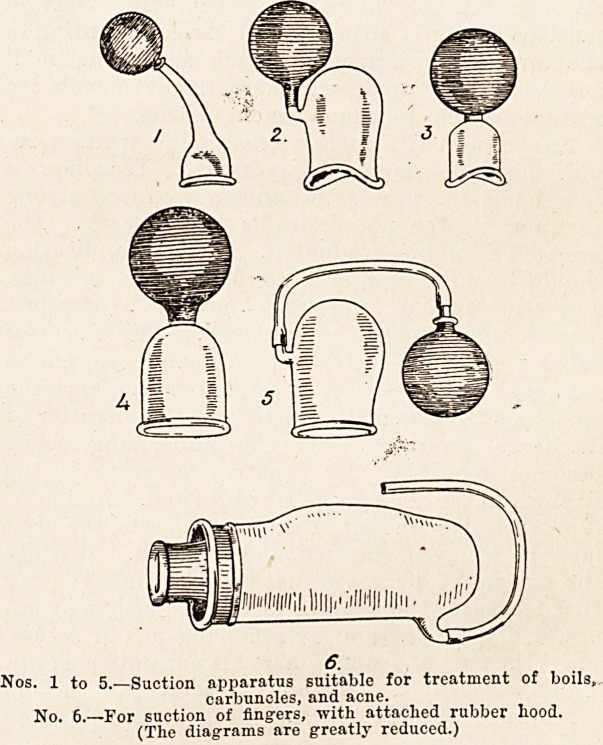# The Treatment of Furunculosis and Suppurative Acne

**Published:** 1907-07-06

**Authors:** William Maclennan

**Affiliations:** Assistant Physician, Western Infirmary, Glasgow; Lecturer, Materia Medica and Therapeutics, Queen Margaret College, Glasgow University; and Clinical Assistant to the Professor of Medicine, Glasgow University


### THE TREATMENT OF FURUNCULOSIS AND SUPPURATIVE ACNE,

### With Special Reference to Bier's Method.

BY WILLIAM MACLENNAN, M.B., Assistant Physician, Western Infirmary, Glasgow; Lecturer, Materia Medica and Therapeutics, Queen Margaret College, Glasgow University; and Clinical Assistant to the Professor of Medicine, Glasgow University.

BIER'S method of treating certain inflammatory affections has recently attracted so much attention, and been employed with so much success, that I think it worth recording my experience of its effects in some common suppurative affections of the skin.

The principle underlying this treatment is not new. Indeed, it is clear that it aims at producing conditions that Nature always, more or less efficiently, inaugurates for herself in every tissue or organ that is injured or that has become the site of infective action. All injured parts become hyperæmic—the hyperæmia being Nature's expression of a process of repair. The redness and swelling are due to an engorgement in the tissues and to an enormous local migration of leucocytes. Bier's treatment aims at reinforcing this natural hyperæmia and enhancing by its means curative influences. It is truly a conservative treatment, the object of which is to prevent, or mitigate, operative interference, and to save mutilation and future loss of function.

Bier employs three methods, or three different forms of appliance, to induce the desired hyperæmia: (1) hot air, (2) a compression bandage, and (3) suction apparatus. All these methods, or appliances, bring about practically the same result. None of them must be used long enough, or strongly enough, to injure the parts, to produce stasis, or to cause pain.

The scope of this paper necessitates only an explanation of the suction apparatus and the technique of its employment. Generally speaking, all superficial circumscribed inflammatory diseases may with great advantage be so treated. Thus carbuncles, boils, and suppurative acne lend themselves peculiarly well to this method, and the treatment of these conditions, with some minor variations in procedure, is also suitable for whitlows, and other surgical septic manifestations of the fingers.

The technique for these cases is simple, yet requires some practice and experience before the best results are attained. The suction apparatus required consists of small glass cups of various sizes and shapes. Their transparency is essential to enable the treatment to be controlled by sight. A convenient set comprises a series of cups ranging from a half to three inches in diameter. For flat and rounded surfaces of the skin the orifices must be of different types, and a variety can be had which makes them adaptable to almost any position. These cups end in a funnel to which is attached, by means of a stout tube, a strong resilient rubber ball for making the suction. The tube is easily detachable, and the whole apparatus can be conveniently sterilised after use. The illustration will show some of the cups which I have found most useful for the treatment of carbuncles, boils, and acne.

Large acne pustules should be opened early, before the skin becomes much implicated, with or without any local anæsthetic, by a simple puncture with a double edged lancet. In this disease the lesions are so numerous that their individual treatment with small cupping glasses would be very tedious. I have found it equally efficacious to employ a comparatively large cup, capable of covering a good area of skin. To expedite treatment these can be applied to several areas of diseased skin at the same séance. The suction for acne should be light and only maintained for three or four minutes at a time. Treatment may with advantage be repeated thrice daily. As strict antiseptic pre-

6.

Nos. 1 to 5.—Suction apparatus suitable for treatment of boils, carbuncles, and acne.
No. 6.—For suction of fingers, with attached rubber hood.
(The diagrams are greatly reduced.)

cautions should be adopted in the treatment of acne as for boils.

In dealing with carbuncles and boils, a slight modification is required according to their stage of development. During the pre-suppurative stage the treatment aims at aborting the inflammatory process so as to prevent the formation of pus. The abortive treatment is carried out as follows.

After cleansing the skin and freezing with ethyl chloride, the thinnest slice—a mere abrasion—of cuticle is removed from the summit of the boil by means of a sharp razor cutting on the flat. No puncture is required, and the minimum amount of local wounding is desirable. The rules to be followed for the application of the suction apparatus are identical with those to be adopted for the second or suppurative stage.

The treatment after pus has begun to form consists in making a free puncture into the centre of the frozen carbuncle or boil. The whole of the cleansed skin surrounding the boil should be smeared with a layer of carbolised lanoline (1 in 40). It is essential to remember this detail, as otherwise the neighbouring skin may readily become infected by the collection of discharge in the suction cup. The lanoline also aids the maintenance of the vacuum and thus serves to keep the cup in position. The suction ball is compressed before its application to the skin, the amount of compression depending on the vacuum desired. On relaxing the pressure, when the cup is in position, it adheres to the greased skin and the tissue bulges into the exhausted cup. When the cup is removed the exudate is to be immediately mopped off and a fresh layer of lanoline applied. Great care must also be taken not to use too small an apparatus, so as to avoid pressure on the surrounding inflammatory area. The suction cup must be large enough to ensure that its edge will only press on healthy skin. Suction must be carefully adjusted and varied according to the various requirements of each case. The small cups commonly used for the treatment of boils are suitable also for the management of acne.

Experience will quickly teach the variation required in the amount of pressure. To compress completely the rubber ball and so produce a strong vacuum is often not desirable or necessary. The pressure should be graduated. The first application should be mild, and, as the inflammation subsides, a greater vacuum, to produce a greater hyperæmia, may be advantageously employed. The object of using a glass cup instead of an opaque apparatus is to enable the operator to estimate at a glance the amount of hyperæmia induced and the quantity of discharge aspirated. If the underlying tissue becomes too livid the negative pressure is too strong and the glass must be readjusted. Similarly, if an excessive amount of exudate collects, the glass must be removed, emptied, and replaced. It is not the amount of discharge that is sucked into the cup that measures the amount of success, although undoubtedly the suction, by removing pus or serum, plays the part of aspiration and is not unimportant. This treatment is not simply a revival of the old "cupping," because, unlike cupping, it is graduated and controlled.

Each treatment should extend over a period of twenty minutes, and at the expiry of each five minutes the cup should be removed for three minutes. At first I tried the application only once a day, but soon I came to find that progress was much more rapid when the séances were shortened and increased in frequency. Thus I would now recommend that two or three times per day the suction should be applied, say, for fifteen minutes at a time, with the removal of the cup each five minutes for a period of three minutes. In dealing with pustular acne the pressure must be light, and the period of application rather—in the first instance at least—curtailed.

I have now treated ten cases of suppurative, or strumous, acne by this method, and in all the improvement was immediate and progressive. The suppurative process was arrested and the scarring diminished. The treatment, too, was a welcome change to the patient from the constant lancing, followed by painful pressure of the pustules—the smallest puncture and the application of the cup constituting now the whole of his local treatment.

I have also tried it, in a large series of boils and carbuncles, with a uniform and encouraging success. A boil has a fairly definite duration, and the time occupied from its inception to its maturation extends from a period of from seven to ten days, or even longer. But an angry boil may be brought to a speedy termination and the suppurative process arrested by the early employment of Bier's method. What is still more, the local pain, and the even worse constitutional disturbance, are often immediately relieved, and the boil heals without the formation of the ordinary core of necrotic tissue.

This method of treatment is very valuable to the practitioner who cannot afford the time of continuous personal superintendence. After some three or four lessons of the management of the apparatus any intelligent patient may be trusted to carry out the procedure without risk of doing harm. Any excess of negative pressure will in most instances proclaim itself to the patient (by pain or discomfort), who will forthwith rectify it by gentle pressure on the ball. The other essential details he will soon pick up. While Bier's treatment is proceeding, constitutional treatment need not be neglected, and the combination of the two will, in my experience, give results that have never hitherto been attained.

The patient should be imbued with the necessity of regarding his affection as one capable of spreading by direct contiguity. He ought to carry out the most thorough cleansing of the skin, both before and after treatment, by the application of spirit, ethereal soap, or other antiseptic. No constant application of ointments is required, and unsightly dressings are discarded. When the suction cup is removed after treatment the exudation spontaneously ceases.

With this treatment, as with all others, success is not invariable, especially till experience is gained, but the results I have obtained convince me of its great superiority over the ordinary methods in vogue. From beginning to end it is practically painless, prevents destruction of tissue and loss of function, leaves a surface which heals without scarring, and almost invariably lessens the duration of these affections.

## Figures and Tables

**Nos. 1 to 5. No. 6. f1:**